# The complex structure of aquatic food webs emerges from a few assembly rules

**DOI:** 10.1038/s41559-025-02647-1

**Published:** 2025-02-28

**Authors:** Ovidio García-Oliva, Kai Wirtz

**Affiliations:** Helmholtz-Zentrum Hereon, Geesthacht, Germany

**Keywords:** Ecological networks, Ecological modelling

## Abstract

Food-web theory assumes that larger-bodied predators generally select larger prey. This allometric rule fails to explain a considerable fraction of trophic links in aquatic food webs. Here we show that food-web constraints result in guilds of predators that vary in size but have specialized on prey of the same size, and that the distribution of such specialist guilds explains about one-half of the food-web structure. We classified 517 pelagic species into five predator functional groups. Most of these follow three prey selection strategies: a guild following the allometric rule whereby larger predators eat larger prey and two guilds of specialists that prefer either smaller or larger prey than predicted by the allometric rule. Such coexistence of non-specialist and specialist guilds independent from taxa or body size points towards structural principles behind ecological complexity. We show that the pattern describes >90% of observed linkages in 218 food webs in 18 aquatic ecosystems worldwide. The pattern can be linked to eco-evolutionary constraints to prey exploitation and provides a blueprint for more effective food-web models.

## Abstract (web summary)

The structure of aquatic food webs does not fit standard allometric predictions. Here the authors explain the co-evolution of specialist and non-specialist predators in aquatic food webs and how this helps reconstruct food webs on the basis of a relatively small number of observations.

## Main

One of the great challenges of ecology derives from the high complexity of food webs^1–3^. The large number of predator–prey relationships makes it extremely difficult to attribute observed changes in single populations to trophodynamics and to devise mechanistic models covering entire (‘end-to-end’) food webs. This difficulty has growing implications because, in particular, aquatic food webs face pressures such as climate change, overfishing and pollution^4,5^. The insufficient realism of ecological models not only limits the ability to estimate the response of aquatic ecosystems to changes in such pressures but also reflects our lack of understanding of the eco-evolutionary mechanisms that shape the structure of (aquatic) food webs. So far, representations of food webs tend to be either overly complex or overly simplistic. The most widespread approach is phenomenological and constructs trophic networks with hundreds or even thousands observed trophic links between individual species or slightly aggregated compartments^6–8^. This approach is missing mechanistic formulations for food-web architecture such as for the sensitivity to environmental stressors^9,10^ and thus cannot easily resolve changes of trophic links in space and time. The second type of ‘end-to-end’ model builds upon size as a fundamental feeding trait to provide a generic and mechanistic but minimal approach to ecological complexity^11–13^. Size-based models were developed on the basis of the allometric rule, which states that larger predators eat larger prey^14,15^. The rule links the size of the most preferred prey, the optimal prey size (OPS, here in units of the equivalent spherical diameter, ESD), with the predator body size^16,17^. However, the allometric rule turns out to be accurate only for a minority of trophic linkages^18^. For example, diverse invertebrate consumers in the 1 mm size class select prey that ranges over three orders of magnitude in ESD^17^.

**Fig. 1:**
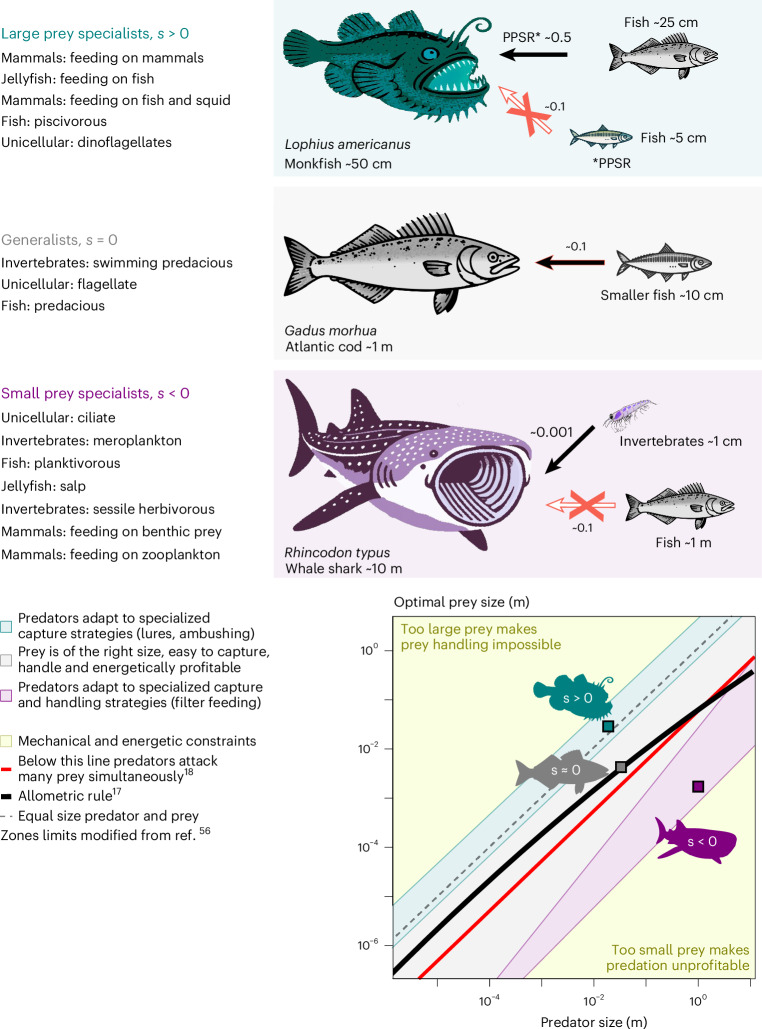
Predator–prey links: actual (black) versus expected (red outline) links based on the allometric rule. Large prey specialists (*s* > 0) select larger (and more profitable) prey than similar-sized predators. Generalist predators (*s* = 0) are mesopredators that select prey proportional to their body size, creating niche differentiation. Small prey specialists (*s* < 0) select smaller (but more abundant) prey than similar-sized predators. Specialized predators compensate physical, that is, energetic and mechanical, limits of prey acquisition, which deviate from the allometric rule. Source data.

Most links that markedly deviate from the allometric OPS rule belong to highly specialized predator guilds, which select prey in a constant and narrow size range despite variations in intraguild predator body size^17,19–23^. This independence from size suggests that also other, complementary traits govern the prey selection of aquatic predators. Here, we introduce specialization as a fundamental trait that can be used to unravel the underlying mechanisms behind the assembly of complex food webs. Specialization quantifies the degree of deviation of the OPS scaling from the allometric rule. The latter can differ across different taxonomic groups. Therefore, our theory aggregates pelagic consumers into predator functional groups (PFGs), which are defined according to similarity in lifestyle traits related to, for example, physiology and life history^24^ (Extended Data Table 1). Predators within a PFG share the size scaling behaviour of the OPS, with the (logarithmic) average value $\overline{\log ({\rm{OPS}})}$. The magnitude of the deviation between PFG-specific $\overline{\log ({\rm{OPS}})}$ and observed OPS provides a quantitative measure for specialization as a numerical trait *s*

1 $$s=\left(\log ({\rm{OPS}})-\overline{\log ({\rm{OPS}})}\,\right)\times {a}^{{\prime} },$$

where ${a}^{{\prime} }$ denotes a PFG-specific normalization constant (Methods). Within each PFG, different values of *s* define distinct predator guilds, which are groups of species with common prey selection strategies in terms of a suite of shared functional and behavioural traits^22^. The value of specialization *s* describes how a guild selects larger (*s* > 0) or smaller (*s* < 0) prey compared with the OPS expected using the allometric rule (*s* = 0). The three constitutive cases are conceptualized in Fig. 1 and outlined in more detail in the Methods. Combining size and specialization as the fundamental traits controlling trophic interactions, we have built a comprehensive, generic and mechanistic theory that explains extremely diverse predator–prey relationships by minimal means. This theory distinguishes three levels of organization: species, aggregated into an individual predator with the species’ mean size, guilds and PFGs—and especially features common characteristics of how specialization of guilds is distributed within PFGs, which can be formulated using few assembly rules. The assembly rules translate PFG- and guild-specific variables into functions that exclusively depend on specialization and the mean size of the guilds and PFGs. These functions, in turn, describe known trade-offs of prey selection such as a lower prey-to-predator size ratio (PPSR) increase with predator size^25,26^. In this study, we used these assembly rules to describe the apparent diversity of whole aquatic food-web architectures and to test our framework in three applications: first, we reconstructed a food-web representation using an extensive dataset of 517 newly compiled observed predator–prey links, aggregated towards OPS and spanning seven orders of magnitude in body size. Second, we tested the usefulness of the theory across a large range of ecosystems and compared predicted and observed predator–prey links for 18 natural sites comprising marine and freshwater food webs. Third, we estimated the minimum number of observed predator–prey interactions required to reconstruct food-web architectures. This number becomes relevant for ecological research in many, yet-understudied habitats, such as in the tropics or the deep ocean.

## Results

### Prey specialization is a widespread trait in aquatic predators

**Fig. 2:**
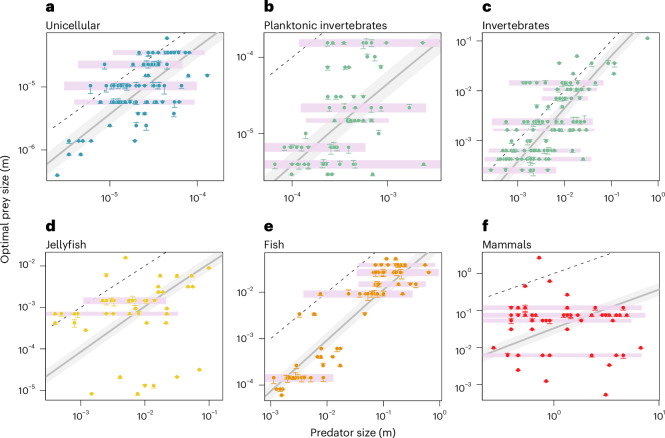
OPS independent from predator size. **a**–**f**, Horizontal bands of constant OPS (pink) are present in all PFGs: unicellular (*n* = 90) (**a**), planktonic invertebrates (*n* = 73) (**b**), invertebrates (*n* = 140) (**c**), jellyfish (*n* = 49) (**d**), fish (*n* = 75) (**e**) and mammals (*n* = 56) (**f**). The displacement in OPS from the observed value (horizontal line) to the centre of the cluster (circle) is displayed as a vertical bar. The grey line shows the OPS expected by the allometric rule specific to each PFG (equation (7) in Methods). The minimum distance between OPS clusters is assumed to be half the general feeding kernel width (indicated by area shading). The 1:1 line is added as dashed line. Data after ref. ^101^. Source data.

To characterize the prevalence of size-independent prey selection in pelagic food webs, we compiled and analysed a dataset of the OPS spectrum across five PFGs: unicellular organisms, invertebrates, jellyfish, fish and mammals (Methods and Fig. 2). Within each PFG, we identified distinct clusters of predators with similar OPS. These clusters are distributed in narrow horizontal bands in the space spanned by body size and OPS, indicating a constant OPS across a wide range of predator sizes (Fig. 2a–f). Many predators in these bands deviate from the allometric OPS rule. For instance, some invertebrates, jellyfish and mammals select prey 100–1,000 times smaller—or larger, in terms of ESD—than what would be predicted for similar-sized predators within their PFG. Additionally, organisms in the 1–10 μm class, typical phytoplankton size, are the preferred prey of a wide range of consumers with ESDs in the range 10^1^–10^5^ μm, thus spanning over 12 orders of magnitude of body volume^27,28^.

**Fig. 3:**
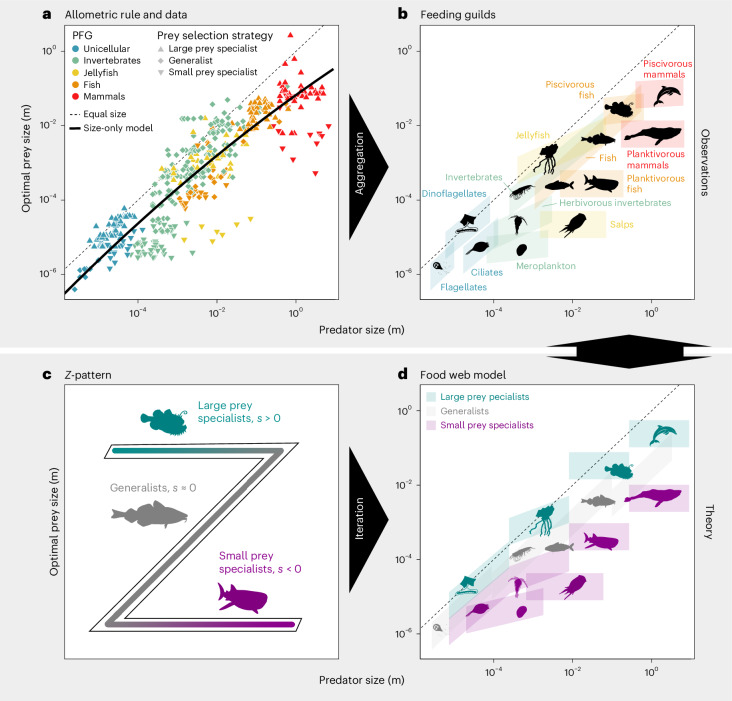
Size-based structure of aquatic food webs. **a**, Trophic links are characterized by the OPS of the predator. The PFGs are colour coded, and prey selection strategies indicated by different point shapes (*n* = 517). The links predicted by the size-only model are depicted as a solid black line (equation (6)). **b**, Feeding guilds aggregated from observed individual OPS links. The height of each box represents twice the width of a typical feeding kernel and covers 95% of the expected predator–prey interactions for each guild, and the width covers the size of all predators assigned to that guild. **c**, Idealized Z-pattern connecting generalists and the specialists to either small or large prey within the fish PFG. **d**, A mechanistic reconstruction of the entire ‘end-to-end’ aquatic food web by iteration and modulation of the universal patterns synthesized from the observations. Data after ref. ^101^. Source data.

Horizontal banding can be formalized and explained by our OPS model: the guild-specific specialization *s* = *s*_*j*_ determined according to equation (1) (see also Methods and ref. ^22^, with index *j* denoting a guild) defines not only the average deviations of the guild from a size-only model (Fig. 3a) but also how the logarithmic size *ℓ* of individual predator species (denoted by index *i*, thus *ℓ*_*i*_) affects logarithmic OPS (${\ell }_{{\rm{opt}}}=\log ({\rm{OPS}})$). Specialization thus changes both the mean size of prey for a guild and the size sensitivity or slope in the size space, respectively (Methods). This slope is 1 at *s*_*j*_ = 0 and very small for ∣*s*_*j*_∣ ≫ 0, thus following the function ${{\rm{e}}}^{-{s}_{j}^{2}}$ so that we have for the OPS

2 $${\ell }_{{\rm{opt}},kji}={C}_{k}+{s}_{j}/{a}_{k}^{{\prime} }+{{\rm{e}}}^{-{s}_{j}^{2}}\times ({\ell }_{i}-{\bar{\ell }}_{k}).$$

**Fig. 4:**
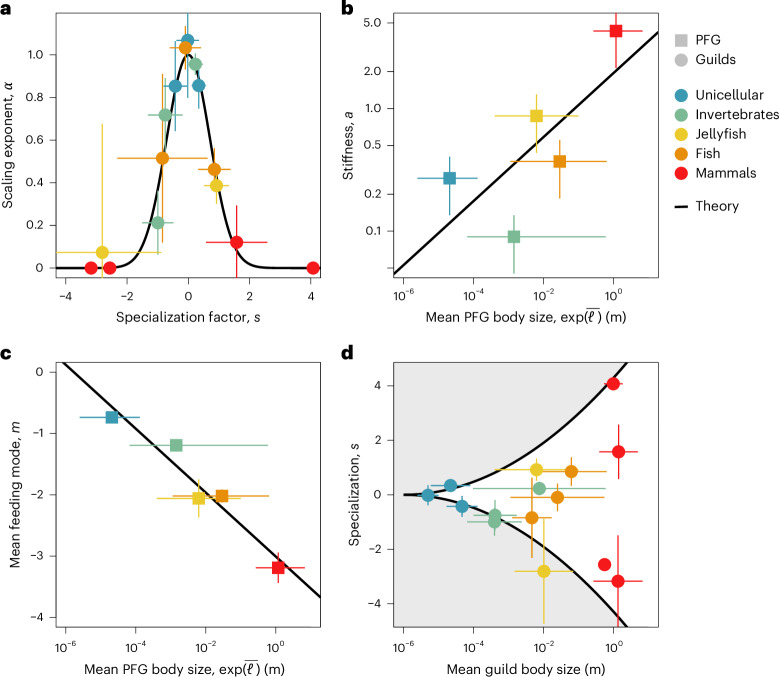
Trade-offs for a mechanistic food-web assembly (see also Table 1). **a**, Nonlinear trade-off between specialization and size-independent prey selection (*n* = 15). The error bars are the standard errors of the scaling exponent (vertical) and the specialization (horizontal). The specific specialization of each PFG was normalized by the square root of its stiffness. Graphs for non-normalized values and stiffness are shown in Extended Data Fig. 1. **b**, Nonlinear trade-off between stiffness, indicating the strength of the specialization in the OPS and reference body size for each PFG (*n* = 5). The error bars are the standard errors of the stiffness (vertical) and the range of predator body size for each PFG (horizontal). **c**, Linear trade-off between the predator–prey ratio and mean predator body size for each PFG (*n* = 5). The error bars are the standard errors of the predator–prey ratio (vertical) and the range of predator body size for each PFG (horizontal). **d**, Nonlinear trade-off between specialization and body size for each predator guild (*n* = 18). The error bars are the standard errors of the specialization (vertical) and the range of predator body size for each predator guild (horizontal). Source data.

Apart from the guild-specific trait *s*_*j*_, all other coefficients in equation (2), that is *C*_*k*_, ${a}_{k}^{{\prime} }$ and the average size ${\bar{\ell }}_{k}$, are common for a PFG *k*. The case ∣*s*∣ ≫ 0 infers size-independent horizontal banding of OPS in specialized guilds. In contrast, at small *s*, OPS follows the allometric rule as visible for a subgroup in, for example, unicellular organisms or fish (Fig. 2a,e). The occurrence of distinct specialization values makes a common pattern across all five PFGs (Extended Data Table 2 and Supplementary Information): the specialization spectrum is subdivided into a positive (8 guilds comprising 153 species, large prey specialists with *s* > 0), a neutral (3 guilds comprising 238 species following the size-only model with *s* ≈ 0) and a negative fraction (7 guilds comprising 87 species, small prey specialists with *s* < 0). Approximately 50% of the species in the dataset are thus classified as specialized predators. The three distinct guilds were found in all PFGs—with few exceptions: the large prey specialists in invertebrates display a specialization *s* only slightly larger than zero, while in two PFGs (mammals and jellyfish), the generalist guilds with *s* = 0 are missing in our dataset. Elsewhere, the three guilds are connected in the space spanned by predator size and prey size, and form an idealized structure resembling a *z* pattern (Fig. 3c and Supplementary Information). This pattern slightly varies among PFGs. To account for these differences, we introduce three non-mechanistic parameters that adjust the orientation, size and positioning of the *z* pattern. These parameters, referred to as rotation, scaling and displacement, give rise to different configurations at the levels of guilds and of PFGs (Methods), as described in Table 1 and illustrated in Fig. 4.

**Table 1:** Four trade-offs identified from our data analysis that reflect inferred eco-evolutionary mechanisms and shape the food-web assembly (for example, by changing the shape of the *z* pattern)

Fig. 3	Related variables	Equation	*r*^2^	Mechanism	Effect on *z* pattern
a	Specialization *s*, size dependency *α*	$\alpha ={{\rm{e}}}^{-{s}^{2}}$	0.97	Complementarity: no predator size dependence of OPS by extreme specialist predators	Rotation: angled (S) to horizontally aligned (L)
b	PFG body size $\bar{\ell }$, stiffness *a*	$a=0.05{{\rm{e}}}^{0.26\bar{\ell }}$	0.53	Constraints (behaviour): specialization impact on the feeding breadth of a whole PFG prey size range	Rescaling: squeezed (S) to expanded (L)
c	PFG body size $\bar{\ell }$, feeding mode *m*	$m=0.12-0.23\bar{\ell }$	0.93	Complexity: larger predators better adapt to smaller prey or increasing volume demand of infrastructure	Displacement: lower Z position for L
d	Predator size *ℓ*, max. specialization ∣*s*∣	∣*s*∣ < 1.9 × 10^−2^ *ℓ*^2^	0.92	Constraints (hydro): lower mechanical constraints at higher Reynolds number	Rotation: angled (S) to horizontally aligned (L)

The strength of each trade-off is evaluated using *r*^2^ from the functional fit shown in Fig. 4a–d. L, large organisms with ESD >1 cm; S, small organisms/PFG with ESD <1 cm.

Source data

To further explore these patterns, we developed algorithmic assembly rules for an idealized food web (Methods and Extended Data Table 3). This model builds on the hierarchy introduced above where coefficients in equation (2) are defined at the PFG level, and on the specialization spectrum described by the *z* pattern for guilds within PFGs, which is modified using emergent trade-offs between PFG- and guild-specific traits (see below). The idealized food web redraws the structured distribution of preferred links collected for the whole spectrum of aquatic consumers (see Fig. 3b for observed guilds and Fig. 3d for reconstructed guilds). It replicates not only the spread of trophic linkages in the predator–prey size space, but also patches with high density of links as matched by artificial guilds—that is, group of predators contained within an algorithmically generated box. Additionally, regions with low density of links are visible as gaps between boxes in our reconstruction. Hence, our model describes the clustering of links in distinct feeding guilds for individual PFGs, while the iteration over PFGs reproduces the overall distribution at the ecosystem scale.

### Food-web assemblies using few assembly rules

**Fig. 5:**
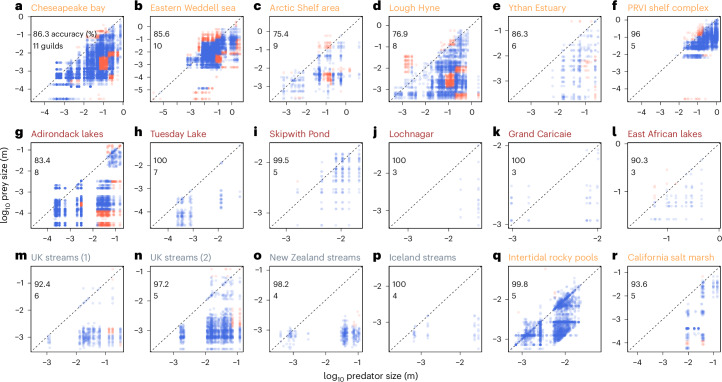
Trophic links in aquatic food webs (*n* = 37,194). **a**–**r**, Reconstruction of aquatic food webs in marine, lake and stream ecosystems (orange, brown and grey labels, respectively), with predator–prey links in terms of their size either predicted (blue) or missed (red) for Chesapeake Bay (**a**), Eastern Weddell Sea (**b**), Arctic shelf area (**c**), Lough Hyne (**d**), Ythan Estuary (**e**), PRVI (Puerto Rico-Virgin Islands) shelf complex (**f**), Adirondack lakes (**g**), Tuesday Lake (**h**), Skipwith Pond (**i**), Lochnagar (**j**), Grand Caricaie (**k**), East African lakes (**l**), UK streams (1) (**m**), UK streams (2) (**n**), New Zealand stream (**o**), Iceland streams (**p**), intertidal rocky pools (**q**) and California salt marsh (**r**). The flexible amount of feeding guilds is inserted in each individual plot. Details on each ecosystem are given in Extended Data Table 4. Data after ref. ^90^. Source data.

However, in real ecosystems, predator–prey links may differ from the idealized case previously discussed (Fig. 3d) as predators consume prey that do not necessarily correspond to their OPS. To evaluate the soundness of our theory in real applications, we applied the food-web model to 18 empirically assembled aquatic ecosystems worldwide (Extended Data Fig. 3). The model accurately reconstructs 92 ± 8% of the observed trophic links with an average of 134 trophic species and 1,689 predator–prey links (Fig. 5 and Extended Data Table 4). This high accuracy is achieved by resolving, on average, 5.9 ± 2.4 artificial feeding guilds characterized by prescribed combinations of size and specialization. These guilds efficiently represent a 20 times higher number of the trophic species observed per food web. Furthermore, we found that spatially open systems, such as estuaries and seas, host more guilds (from 5 to 11; Fig. 5) than more closed systems, such as streams and lakes, entailing fewer guilds (3–8).

**Fig. 6:**
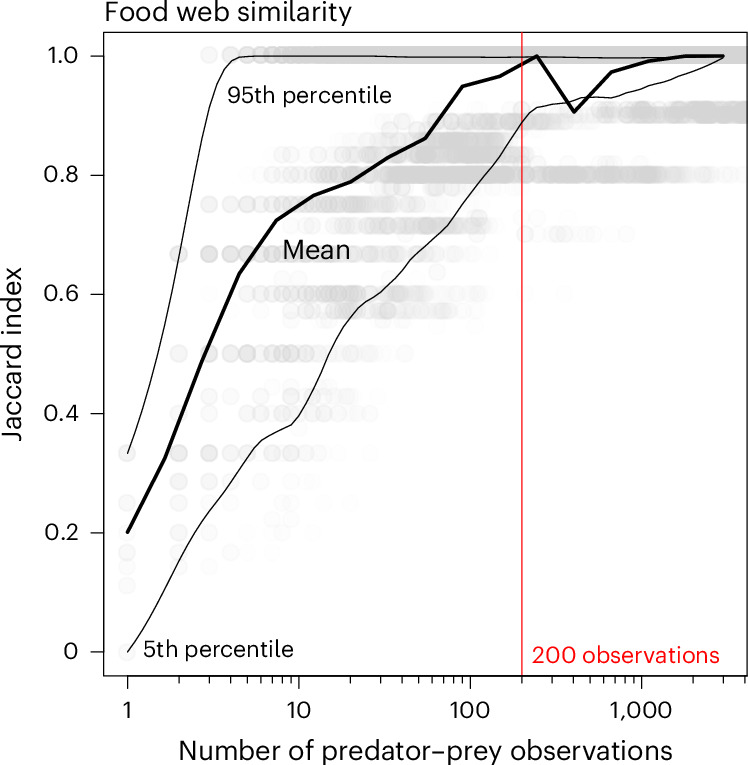
Comparison of food-web reconstructions based on the full datasets or on smaller subsamples (*n* = 32,104). As a quality assessment of the reconstructions, we used the Jaccard similarity index (Methods). A minimal number of observations—approximately 200, representing just 10% of the mean observations in empirically assembled food webs (Fig. 5 and Extended Data Table 4)—is sufficient for accurately reconstructing pelagic ecosystem food webs. Source data.

We tested the potential of our model to reconstruct food webs under restricted data availability. Our results show that approximately 200 predator–prey observations are sufficient to accurately reconstruct food webs while preserving their structure (Fig. 6). In each reconstruction, the model was applied to a randomized subsample of observed predator–prey pairs, and the resulting assembly was compared with the original food web using the Jaccard similarity index (Methods). We found a high similarity between the reconstructed food webs that rely either on around 200 or on all available observations, even if these exceed several thousand links (Supplementary Information). This result demonstrates the feasibility of assessing the complete structure of a food web with a relatively small number of known trophic links, making the model valuable in situations where data are limited.

## Discussion

### Eco-evolutionary origin of specialization

Our results highlight that size-independent prey selection is a prevalent trait among aquatic consumers, transcending taxonomy and lifestyle. Prey specialization appears widely across our dataset, which indicates its fundamental role in aquatic food webs and that it is subject to a series of common processes independently of taxonomy and habitat characteristics. Changes in the distribution of specialization in turn substantially shape food-web structures. These changes occur on different timescales: (1) ecological, such as reflecting shifts in predator and prey abundances; (2) evolutionary, by selecting successful predator and prey traits; and (3) eco-evolutionary, where ecological and evolutionary processes influence each other over time^29^.

The ecological importance of specialization as a fundamental trait appears in analysing food webs under variable prey abundance^30^. In ecosystems with high variability in prey availability, specialization can pay off by exploiting an abundant resource, such as phytoplankton, or reducing competition due to resource partitioning^30,31^. Despite the broad range of their body sizes, specialized predators compete for prey in a restricted size range. Thus, these predators are often efficient in acquiring prey, such as large prey specialists observed in ecosystems with low prey density^32^, at the top of the food web^33^, in social predators^34^ and in predators with high intraguild predation^32,35^. Social predation marks a strategy especially promoting specialization, as swarming animals are more successful in hunting down large prey or can trap many small organisms^36,37^. Competition of specialized predators at ecological timescales can also be sustained by high prey abundance. In particular, small prey specialists access a larger resource pool since smaller organisms generally dominate the biomass spectrum^38^.

At evolutionary timescales, the distribution of specialization changes due to, for example, foraging adaptation or the development of specialized traits for specific prey acquisition^39^. As the relative fraction of small prey increases with the habitat size and productivity^40^, large filter feeders (*s* ≪ 0) evolutionary appeared after large increases in primary production, hence of prey density^39,41^. Predator species evolve specialized traits to capture specific prey types more efficiently^42,43^. Prey species, in turn, build defences to avoid predation, generating a co-evolutionary arms race^44,45^. In response, predators may adapt by further specializing in the prey type that is more abundant or accessible (for example, refs. ^44,46^). The evolutionary arms race takes place in ecosystems that typically display high spatial and temporal variability^47^, especially in the distribution and abundance of prey^48^.

Evolutionary changes affect ecological processes and patterns and thus lead to interactions between ecological and evolutionary processes in an eco-evolutionary dynamic^29^. For example, different prey specialization patterns originate from the feedback between ecological conditions and evolutionary processes, such as convergent and diverging evolution. During convergent evolution, different taxa evolve similar traits in response to similar ecological stimuli^49–51^. Despite their different feeding mechanisms, evolutionary origins, body sizes and association to PFGs, dinoflagellates^22^, salps^23^ and meroplankton^27^ have all specialized in the often dominating phytoplankton 1–10 μm size class^52^. This convergent prey selection is possible only in cases where this prey size is indeed abundant, which depends on ecological conditions such as biomass and productivity^38,41,53^. In contrast, parallel evolution splits a monophyletic taxon into branches with different traits specialized for a specific diet that creates niche differentiation, such as for cetaceans^54^, siphonophores^21,32^ or dinoflagellates^22^. In either case, the advantages of adaptations towards specialization may still be restricted by evolutionary constraints that conserve ecological interactions^45,55^ and physical and energetic limits of the predator–prey interactions^56,57^.

The success of specialized predators indicates that some species have developed strategies to compensate for the physical—that is, energetic and mechanical—limits of prey acquisition, which can be expressed as a set of allometric rules^56^ (Fig. 1). For example, the size of the consumed prey limits the net energy gain of a predator: too small prey may not provide enough energy, whereas too large prey may require too much energy to capture. In both cases, the predator experiences a negative energy balance. Allometric rules for OPS thus describe predator–prey interactions that provide a net energy gain for the predator^56,58^, which in turn confines the size of the preferred prey. Owing to the energy demand and mechanical limitations of capturing larger organisms^42,59^, preference towards large prey requires morphological and behavioural adaptations such as ambush foraging, toxins, specialized grabbing appendages or complex predation techniques including cooperative hunting and ambushing^43,60^. By contrast, the acquisition of small prey is restricted by the energetic costs of individual prey capture and handling^56^. Capturing small prey with a net energetic gain thus requires specialized handling strategies such as filter feeding or consumption of several prey items at a time^41,61^. With these adaptations, specialized predators acquire prey unfavourable to similar-sized predators, creating niche differentiation and promoting species coexistence^62^, but also drive nonadapted species to extinction^63^, such as experienced, for example, by early cetaceans^54^.

### Trade-offs between aspects of specialization and size

The physical and eco-evolutionary constraints of predator–prey interactions can be thought to be inherent to the four essential trade-offs formalized with our framework. The first trade-off (*α* versus *s*) links extreme cases of specialization—towards very small or very large PPSRs—to prey selection that becomes size independent (Fig. 4a). Size-independent prey selection appears in, for example, filter feeders (small prey specialists with *s* ≪ 0), which capture prey using sophisticated structures that are not—or are just marginally—correlated with body size, such as jaws in rotifers^64^, setulae (distance) in cladocerans^65^ and baleen in whales^19^. The strength of the trade-off involving increasing effects of specialization on the OPS scaling is measured by the stiffness (*a*). The stiffness can be interpreted as the inverse feeding breadth of a PFG since its value determines the range of guild-specific OPS within a PFG (cf. equation (11) and Supplementary Information, equations A9 and A10). This inverse feeding breadth was here found to depend on the mean PFG body size, as larger predators tend to exhibit stiffer prey selection (Fig. 4b). For example, invertebrates feed on a broader size range than mammals, although the latter possess more extreme specialization values than invertebrates. We explain this difference by a less diverse taxonomy of larger PFGs (see Supplementary Data 1 for the taxonomy of the included species), limiting their access to a wider spectrum of prey size. The weak correlation between stiffness and mean body size of PFGs, especially for the smaller ones, suggests that factors beyond size, such as metabolism or movement type (for example, ref. ^26^), play an important role. These factors are more varied among smaller PFGs, which show a more diverse array of traits^57^.

Body size still plays a role in limiting the OPS as its increase reduces the PPSR (Fig. 4c). This trade-off has been found before (for example, refs. ^25,26,66^) and was attributed to an increased demand of intrabody transport networks with body size^17^ and to nonlinear size dependencies of metabolic, structural and morphological traits involved in prey acquisition (for example, ref. ^67^). Finally, body size also constrains the range of available specialization values (Fig. 4d). In planktonic organisms, the maximum absolute value of specialization increases with body size. This trend weakens for predators in the plankton–nekton transition and is lost in swimming predators (size >7 cm (ref. ^24^)). We explain this pattern with the hydrodynamics of predator–prey interactions: predators exploit enhanced encounter rates for particles in the intermediate Reynolds regime (approximately 0.1 mm, 0.1 ≲ Re ≲ 50), which maximizes prey capture^68^. The hydrodynamics of prey capture might also explain the dominance of negative specialization values among smaller PFGs, which display even more than one negative specialization and thus a prominent role of filter feeding (Fig. 3d). These hydrodynamic effects are more compelling for plankton predators^57^ so that they can be expected to be less important for swimming predators at higher Re (ref. ^24^).

### Simplified assembly of complex food webs

The expression of universal rules behind prey selection with our specialization theory enables us to devise end-to-end food-web models using minimal parameter configurations. The assembled models describe trophic relations for nearly the complete body size spectrum, from nanoflagellate grazers to whales, with minimal input of six generic parameters—that is, five fitted parameters for the trade-off equations (Fig. 4) and a size class width (Supplementary Information). The flexible amount of feeding guilds is determined by an algorithm that maximizes the coverage of links in the space of predator size and prey size (Extended Data Table 3). This approach demonstrates very high skill in food-web reconstructions for diverse marine and freshwater ecosystems with multiple levels of complexity^26^. While our model is accurate at 92% by assuming a unique parameter set, other detailed mechanistic theories for food-web representation, such as the allometric diet breadth model (accuracy <65% (ref. ^58^)) or the individual-based food-web model (83%; refs. ^69,70^) require specific parametrizations for each food web. More general mechanistic approaches with a similar number of parameters resolve much less trophic links within aquatic food webs compared with our approach (92%), such as the size-constrained feeding-niche model reproduces 68% (ref. ^71^) and the size-only model 60% (equation (6) and Extended Data Fig. 4). In each of the food-web reconstructions, only the number of feeding guilds differs, as certain guilds are absent in some ecosystems such as large mammals lacking in lakes and streams. The food-web assemblies thus emerge from the combination of feeding guilds and their trophic relationships.

The high similarity of reconstructed and observed food webs (Fig. 6 and Supplementary Information) indicates that the eco-evolutionary rules embedded in our model enable robust food-web assembly, even with a limited number of observations. It highlights the value of our theoretical approach for assessing understudied food webs with limited access to direct observations, for example, deep pelagic ecosystems^72^. In addition, by transferring knowledge about food-web structures from well-studied to understudied locations, our model might open new opportunities to utilize datasets with reduced information about predator–prey interactions but rich observed individual size data, for example, refs. ^73,74^. Summing up, we present a set of rules to describe food-web assemblies, which allows mechanistic (that is, physical and eco-evolutionary) interpretations and is simple and accurate at the same time.

The specialization theory may hence much improve the representation of trophodynamics in ecological models. For example, popular phenomenological models require hundreds of parameters obtained from sampling to describe local food webs, for example, refs. ^9,75^, which challenges their spatial application or their extrapolation in time^7,76^. Conversely, models built upon size as the fundamental and unique feeding trait provide a simplified approach to food-web structure^13,77,78^; however, at the cost of low accuracy (Extended Data Fig. 2). The few modelling strategies combining both approaches—that is, functional groups plus size-based interactions—still rely upon many coefficients inherent to prescribed trophic links to describe food-web structures (see, for example, refs. ^79,80^). These limitations impede the understanding of aquatic ecosystems since most models seek to reduce food-web complexity by focusing either on higher or on lower trophic levels, which creates a biased perspective on food-web functioning as either bottom-up (that is, in biogeochemical and nutrient–plankton models) versus top-down dominated (for example, fisheries models)^81^. A seamless continuation of trophic levels without an imposed top-down/bottom-up perspective will produce a better understanding of changes in food-web functioning. The latter is a key requirement in assessing current and predicting future ecosystem responses (see, for example, refs. ^16,82^).

Our study has unravelled a fundamental pattern in the selection behaviour of aquatic predators. By including size-independent prey selection, we devised a food-web model that reproduces multiple ecosystems with high accuracy. As a second fundamental feeding trait in addition to size, the specialization trait offers a framework to identify and quantify universal laws in food-web assemblies. This finding may guide empirical assessments, also with respect to optimal aggregation levels of ecological variables. The universality of the observed prey selection strategies suggests that prey specialization results from deeper ecological drivers as part of eco-evolutionary processes. Our approach creates a blueprint for end-to-end food-web assemblies with improved accuracy while providing a simple framework for the analysis of food-web responses to multiple stressors^4^ such as global warming^83,84^, overfishing^85^ and other changes in the oceanic environment^5,9,86^.

## Methods

### Data integration

We compiled a dataset of the OPS of pelagic planktonic^17,22,27,87^ and nectonic^88^ predators. As OPS of fish and invertebrates were not readily available, these were estimated from an extensive selection (*n* = 54,635) of predator–prey links reported by ref. ^89^ for fish, and by refs. ^18,90^ for invertebrates. For both PFGs, the mean predator and prey sizes were selected as the predator–prey link representative for each species only if (1) the predators were identified to a species level and (2) more than five observations have been reported, which resulted in 517 predator species. For each species, detailed taxonomic information was extracted from the World Register of Marine Species (https://www.marinespecies.org/, accessed 9 March 2023). All sizes are expressed as ESDs in metres.

### Horizontal bands in the OPS spectrum

We define horizontal bands as clusters of OPSs, whose means are separated by a minimum distance forming a gap between them. We performed a sequential hierarchical cluster analysis^91^ by reducing the number of clusters until the minimum distance between the mean log-size of consecutive clusters was $> \frac{1}{2\sqrt{3}}$ (approximately the universal half-width of the feeding kernel in units of logarithmic ESD^92^).

### Prey selection strategies within PFG

In our analysis, predator–prey links are averaged for individual predator species (neglecting variable sizes within a predator population) and concentrated to the most preferred prey size (OPS), thus neglecting the typically broader size range of actually ingested prey. These aggregated predator–prey links are represented by an OPS allometric rule, which depends on predator body size expressed as its ESD. Note that our usage of ESD emphasizes the body volume and mass of the feeding and fed organisms, while one-dimensional characteristics, such as body width or length, are neglected due to the highly diverse aspect ratios of aquatic organisms.

The relative importance of the predator body size compared with other non-size traits is quantified by a variable OPS scaling exponent *α* via the allometric relation

3 $${\rm{OPS}}\propto {{\rm{ESD}}}_{{\rm{predator}}}^{\alpha }.$$

This relation is better represented using log-transformed variables, which simplify the mathematical relationship and enhance interpretability, as

4 $${\ell }_{{\rm{opt}}}\propto \alpha \times \ell ,$$

where $\ell =\log ({\rm{ESD}}\,{{\rm{m}}}^{-1})$ and ${\ell }_{{\rm{opt}}}=\log ({\rm{OPS}}\,{{\rm{m}}}^{-1})$ are the logarithms of the predator body size and the OPS, respectively.

The log-transformation linearizes the relationship between predator and prey. When the body size is crucial for prey selection, the dependence of OPS on predator size equals a linear size scaling (*α* = 1). On the contrary, when body size does not influence prey selection, *α* = 0 describes size independence of OPS, in which only behavioural and non-size traits linked to predator lifestyle constrain the OPS^14,24,26^. The scaling exponent *α* thus quantifies the relative weight of individual size in prey selection and its complement (1 − *α*) quantifies the relative weight of non-size traits in prey selection. The combination of both aspects yields

5 $${\ell }_{{\rm{opt}}}\propto \alpha \times \ell +(1-\alpha )\times \bar{\ell },$$

where $\bar{\ell }$ is a reference body size that encapsulates non-size-related feeding traits.

According to its definition^22^, $\bar{\ell }$ is the average body size of predators that share similar lifestyle and non-feeding-related traits and serves as a baseline for size-independent aspects of predation. Even when prey selection is entirely size independent (*α* = 0), the OPS remains constrained by the size of this idealized predator. This concept aligns with taxonomy as a key factor in prey selection^93,94^, along with influences of other factors such as prey availability, habitat, consumer type and metabolic type^18,26,50,88,95–97^. We classify predators into five functional groups (PFGs) based on fundamental physiological and life history traits: (1) unicellular organisms, (2) invertebrates, (3) jellyfish, (4) fish and (5) mammals. Four of these groups (1, 2, 4 and 5) were already categorized as unicellular mixotrophs and heterotrophs, planktonic multicellular heterotrophs with ontogenetic growth, visually foraging poikilotherms and homeotherms, respectively^24^.

Equation (5) serves as the foundation for a new OPS-scaling rule, which we outline in the following sections.

### Specialization model

OPS has already been formulated as a function of individual predator log-size *ℓ*_*i*_ (ref. ^17^) as

6 $${\ell }_{{\rm{opt}},i}^{\dagger }=r+{\ell }_{i}-\gamma \times {\ell }_{i}^{2},$$

where ${\ell }_{{\rm{opt}},i}^{\dagger }$ is the OPS of a predator of size *ℓ*_*i*_ according to scaling^17^, *r* is the prototype scaling—that is, the logarithmic PPSR averaged over all aquatic predators—and *γ* is a structural coefficient that quantifies the decline in the slope of the OPS due to the augmented volume demand by the internal transport network of an enlarged body^17^.

The PPSR has been found to vary among PFGs^93,94^. This change in the ‘offset’ OPS can be expressed by the size-independent feeding mode *m*, which quantifies the relative activity—that is, active ‘raptorial’ or passive ‘filtration’—of the feeding strategy of a PFG without any allometric effect^17^. Passive strategies (no or slow swimming or suspension feeding) are characterized by a negative feeding mode, while directional ambushing and chasing by *m* > 0. The feeding mode vanishes (*m* = 0) for a neutral or mixed strategy. Numerically, the feeding mode is the change of the PPSR relative to the prototype scaling *r*. Let the index *k* denote the PFG, then the insertion of PFG-specific characteristics such as offset *m*_*k*_ and minimum body size ${\ell }_{k}^{{\prime} }$ into equation (6) yields a second OPS model

7 $${\ell }_{{\rm{opt}},ki}^{\ddagger }=r+{m}_{k}+{\ell }_{i}-\gamma \times {({\ell }_{i}-{\ell }_{k}^{{\prime} })}^{2},$$

where ${\ell }_{{\rm{opt}},k}^{\ddagger }$ is the allometric scaling adjusted for the PFG *k*. In this case, the minimum body size accounts for the difference in morphology and transport network topology among PFGs. For example, the minimum body sizes and, in consequence, the volume demands by the internal transport network of an enlarged body, are different for unicellular predators than for mammals^98^.

From equation (7), we construct an OPS scaling model that accounts for variations of the PPSR within the same PFG. Deviations from the average OPS scaling of a PFG are here quantified using the specialization *s* (Extended Data Fig. 5). The specialization trait defines how the PPSR changes in relation to the feeding mode *m* of generalist predators. If *s* = 0, the feeding mode of a guild is identical to the generalist type, while *s* > 0 and *s* < 0 indicate that the effective feeding mode *m* + *s* increases or decreases, respectively. Hence, *s* captures deviations in the feeding mode *m* (and resulting OPS) from generalist guilds to specialized guilds feeding in both directions of the prey spectrum to smaller and larger organisms^22^.

The value of the specialization of a feeding guild *j*, termed *s*_*j*_, is calculated proportional to the difference between the log-OPS calculated using allometric scaling adjusted for a PFG (equation (7)) and a linear regression of the log-OPS as function of the predator log-size

8 $${\ell }_{{\rm{opt}},kji}={\alpha }_{kj}\times {\ell }_{i}+{m}_{kj}^{{\prime} },$$

where ${m}_{kj}^{{\prime} }$ denotes the intercept and *α*_*kj*_ a scaling exponent specific for a feeding guild *j* within a PFG *k*. The difference between equation (8) and equation (7) is thus proportional to the specialization value^22^

9 $${s}_{j}=\left({\ell }_{{\rm{opt}},kji}-{\ell }_{{\rm{opt}},ki}^{\ddagger }\right)\times {a}_{k}^{{\prime} },$$

where ${a}_{k}^{{\prime} }$ is a provisional and PFG-specific proportionality constant. After simplification by evaluating the expression at the reference body size ${\ell }_{i}={\bar{\ell }}_{k}$ to remove the effects of variations in predator size, the definition of specialization reads

10 $${s}_{j}=\left({m}_{kj}^{{\prime} }-r-{m}_{k}-(1-{\alpha }_{kj})\times {\bar{\ell }}_{k}\right)\times {a}_{k}^{{\prime} }.$$

Note that, by evaluating equation (10) at the reference body size, specialization equals the difference in the OPS of a specialized predator and a predator that follows the allometric rule (*s* = 0 and *α* = 1). See Extended Data Fig. 5 for an applied example of this interpretation.

Inserting equation (10) into equation (7) yields the specialization model

11 $${\ell }_{{\rm{opt}},kji}=r+{m}_{k}+{s}_{j}\times {a}_{k}^{-1/2}+{\bar{\ell }}_{k}+{\alpha }_{kj}\times ({\ell }_{i}-{\bar{\ell }}_{k})-\gamma \times {({\ell }_{i}-{\ell }_{k}^{{\prime} })}^{2},$$

where *α*_*kj*_ is a positive value that quantifies the strength of the link between OPS and prey specialization for a PFG, which we term stiffness^22^. In this case, ${a}_{k}^{1/2}$ replaces the constant ${a}_{k}^{{\prime} }$ and is derived from a normalization scheme described in the Supplementary Information. Low values of *a*_*k*_ describe large shifts of OPS even for small changes in *s* to smaller (*s* < 0) or larger prey (*s* > 0). High values of *a*_*k*_ reduce the dependence of the OPS on *s*, so that the difference on OPS between generalists (*s* = 0) and specialists (*s* ≠ 0) vanishes at small variations of *s*.

### Specialization and size scaling

In equation (11), specialization *s* quantifies an offset in the OPS. In addition, specialization is here assumed to mediate the dependence of OPS on predator size as represented by the exponent *α* (equation (5)). By construction, the exponent *α* equals 1 for pure generalist non-specialized predators with *s* = 0. With increasing specialization (∣*s*∣ ≫ 0), the OPS size dependence and thus the exponent *α* should vanish. A simple functional relationship *α*(*s*) meeting the two demands is

12 $$\alpha (s)={{\rm{e}}}^{-{s}^{2}}.$$

Equation (12) represents a trade-off between size-dependent prey selection and a PPSR that deviates from the PPSR of generalist predators (Fig. 1). The combination of this relation with the specialization model yields the simplified equation

13 $${\ell }_{{\rm{opt}},kji}={C}_{k}+{s}_{j}\times {a}_{k}^{-1/2}+{{\rm{e}}}^{-{s}_{j}^{2}}\times ({\ell }_{i}-{\bar{\ell }}_{k}),$$

where *C*_*k*_ is a constant within a PFG and formally defined as ${C}_{k}\approx r+{m}_{k}+{\bar{\ell }}_{k}$.

Highly specialized guilds have ∣*s*_*j*_∣ ≫ 0, so that the exponential term vanishes (as e^−*∞*^ = 0) and OPS in equation (13) becomes constant

14 $${\ell }_{{\rm{opt}},kji}={C}_{k}+{s}_{j}\times {a}_{k}^{-1/2}.$$

This OPS is independent of the predator body size *ℓ*_*i*_.

On the contrary, for generalist guilds with *s*_*j*_ = 0, the exponential term converge to one (as e^0^ = 1) and yields a linear size-dependent OPS similar to equation (7),

15 $${\ell }_{{\rm{opt}},kji}={C}_{k}+{\ell }_{i}-{\bar{\ell }}_{k}.$$

### Observed food-web reassemblies using a mechanistic model

For constructing predator–prey links as foundational elements of efficient food-web models, we describe each feeding guild by mean body size and a specialization value. We propose a set of assembly rules to assign body size and a specialization value that minimizes the overlap between feeding guilds while maximizing the coverage of links in the space of predator size and prey size, thus introducing a new size class immediately after the anterior with no gaps in between. Each size class covers a specific region of the predator size spectrum^99^ of *w* = 3.4 units in log size (Supplementary Information) and includes four specialization values for planktonic predators (one positive, one neutral and two negatives) and three for nektonic predators (one positive, one neutral and one negative). Specialized guilds with *s* ≠ 0 are in addition displaced by one log-size unit—approximately a factor of 3—to minimize the overlap with generalists in the lower and upper ends of the *z* pattern. This choice minimizes redundant guild assignation to predators that may lie in either category and illustrates niche differentiation. We assume a minimum predator size of a heterotrophic nanoflagellate around 2.5 × 10^−6^ m, so that four size classes are needed to cover the entire predator size spectrum with a maximum body size of 10 m. The middle point of each size class is the reference body size, which is the value utilized in the trade-off equations (Table 1 and Fig. 4a–d). An algorithmic representation of the procedure is described in Extended Data Table 3.

This mechanistic scheme to represent food-web structure was applied to 18 sites covering marine, estuarine and freshwater ecosystems (*n* = 37,194)^90^. Each observed predator–prey link in these sites was matched to the closest size-OPS pair predicted by our model. When the deviation between observed and predicted predator–prey links was less than $\frac{2}{\sqrt{3}}$ (approximately a factor of 3 in the linear OPS, twice the universal width of the feeding kernel that covers 95% of the ingested prey^66,92^) the observation was tagged as correctly predicted. Otherwise, it is labelled as a missed link. The model accuracy is defined as the percentage of correctly represented predator–prey pairs in each ecosystem. The model includes the feeding guilds that cover correctly predicted links.

### Food-web similarity with reduced observations

To evaluate the robustness of our model in characterizing food-web architectures, we reconstructed food webs using a reduced number of observations. Through a series of Monte Carlo experiments (with *n* = 32,104 iterations), we applied our model to randomly selected observed predator–prey pairs in each of the 18 analysed ecosystems. Subsamples varied in size, ranging from single observations to complete experimental datasets.

We used the Jaccard similarity index *J* (ref. ^100^) to compare the co-occurrence of feeding guilds between food webs reconstructed using (1) the complete set of observations—list *R*, of reference—and (2) a subset of observations—list S, of subsample. Each list, *R* and *S*, contains 14 binary attributes, corresponding to each potential feeding guild shown in Fig. 3d. These attributes are binary, representing the ‘absence’ (0) or ‘presence’ (1) of a feeding guild. The Jaccard similarity index *J* measures the overlap between the attributes of *R* and *S*. A Jaccard similarity index value close to one indicates a high degree of similarity between the food web obtained from the subset of observations (list *S*) and the reference food web (list *R*). The reference food web represents the most faithful representation possible using of the empirical food-web assembly. In other words, the reconstructed food web closely resembles the empirical food-web assembly (Supplementary Information), suggesting that the model accurately captures the underlying patterns of predator–prey interactions.

The Jaccard index is calculated as

16 $$J=\frac{{x}_{11}}{{x}_{01}+{x}_{10}+{x}_{11}}.$$

*x*_11_ represents the total number of guilds where *R* = 1 and *S* = 1, *x*_01_ is the total number of attributes where *R* = 0 and *S* = 1, and *x*_10_ is the total number of attributes where *R* = 1 and *S* = 0.

### Reporting summary

Further information on research design is available in the Nature Portfolio Reporting Summary linked to this article.

## Supplementary information

Supplementary Information Supplementary Methods 1–4 and Figs. 1–4.

Reporting Summary

Supplementary Data 1 Taxonomy of included species.

## Source data

Source Data Fig. 1 Data digitized from ref. ^56^ available at https://doi.org/10.1111/1365-2435.13254.

Source Data Fig. 2 Generated by script Data-processing-1.R.

Source Data Fig. 3 Generated by script Data-processing-1.R.

Source Data Fig. 4 Generated by script Figure-Script-1.R.

Source Data Fig. 5 Generated by script Figure-Script-2.R.

Source Data Fig. 6 Generated by script Figure-script-8.R.

Source Table 1 Generated by script Figure-Script-1.R.

**Extended Data Fig. 1:**
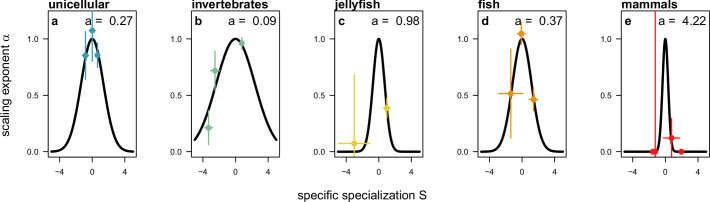
Link between size-dependence and prey specialization for each PFG. Strength of the trade-off between the scaling exponent *α* and specific specialization *S* as described by the stiffness *a* for all five PFGs (**a**) unicellular organisms (*n* = 91), (**b**) invertebrates (*n* = 246), (**c**) jellyfish (*n* = 49), (**d**) fish (*n* = 75), and (**e**) mammals (*n* = 56). A simple trade-off function $\alpha (S)={{\rm{e}}}^{-{(a\cdot S)}^{2}}$ fulfilling the requirements *α*(0)=1 and *α*(± *∞*)=0 is drawn in black. Standard errors of the scaling exponent and the specific specialization are given as vertical and horizontal error bars, respectively. The normalization *s* = *S* ⋅ *a*^1/2^ enables a direct comparison of specific specialization values of all PFGs in Fig. 4 using the function $\alpha (s)={{\rm{e}}}^{-{s}^{2}}$. Source data

Source Data Extended Data Fig. 1 Generated by script Figure-Script-1.R.

**Extended Data Fig. 2:**
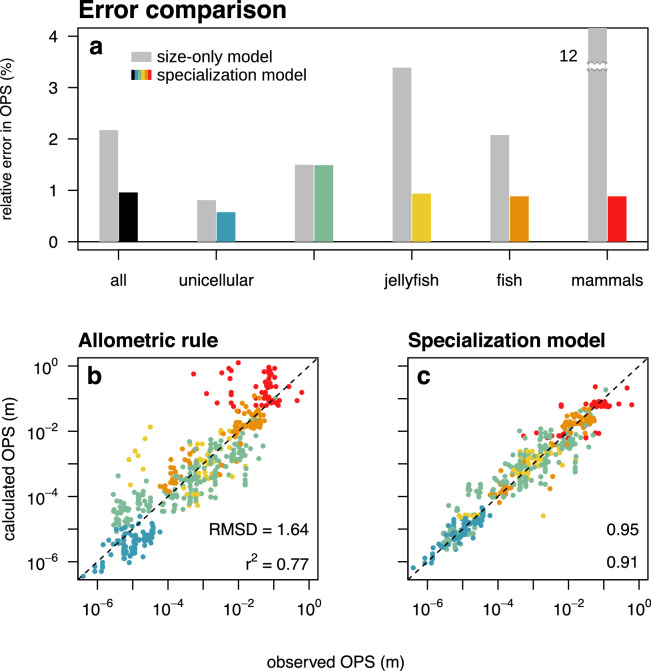
Error in the OPS estimation. (**a**) The relative errors of the estimated OPS ($=| \frac{{\rm{observedOPS}}-{\rm{estimatedOPS}}}{{\rm{observedOPS}}}|$) were calculated (*n* = 517) using the allometric rule (gray bars) and the specialization model (colour bars). The bar covers 80 percent of all predators, in black, as well as for each PFG. (**b**) The calculated vs. observed OPS is compared for the size-only model, and (**c**) for the specialization model. The root-mean-square deviation (RMSD) and the coefficient of determination (*r*^2^) are given for each model. Data after ref. ^101^. Source data

Source Data Extended Data Fig. 2 Generated by script Figure-Script-1.R.

**Extended Data Fig. 3:**
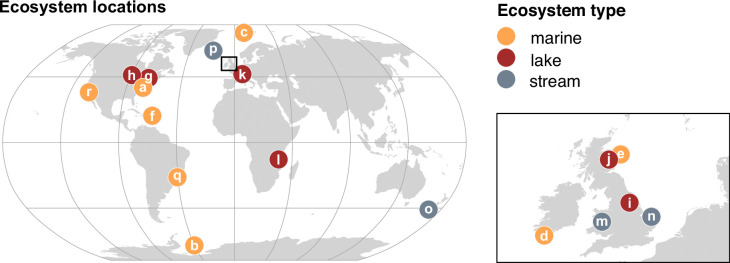
Location and ecosystem type of the food-webs represented by our model (*n* = 18). see also Extended Data Table 4. Data after ref. ^90^. Basemap from Natural Earth (https://www.naturalearthdata.com). Source data

Source Data Extended Data Fig. 3 Map coordinates in ref. ^90^ available at https://doi.org/10.25829/iDiv.283-3-756.

**Extended Data Fig. 4:**
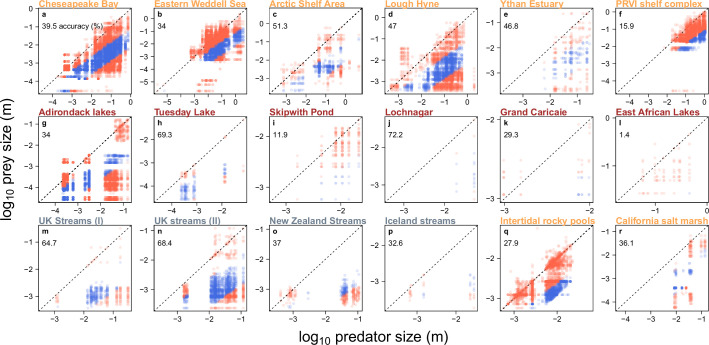
Aquatic food-webs (*n* = 37, 194). Reconstruction of aquatic food-webs using a size-only model in marine, lake, and stream ecosystems (orange, brown, and gray labels, respectively), with predator–prey links either predicted (blue) or missed (red) for Chesapeake Bay (**a**), Eastern Weddell Sea (**b**), Arctic shelf area (**c**), Lough Hyne (**d**), Ythan Estuary (**e**), PRVI (Puerto Rico-Virgin Islands) shelf complex (**f**), Adirondack lakes (**g**), Tuesday Lake (**h**), Skipwith Pond (**i**), Lochnagar (**j**), Grand Caricaie (**k**), East African lakes (**l**), UK streams (1) (**m**), UK streams (2) (**n**), New Zealand stream (**o**), Iceland streams (**p**), intertidal rocky pools (**q**) and California salt marsh (**r**). The flexible amount of feeding guilds is inserted in each individual plot. Details on each ecosystem are given in Extended Data Table 4. Data after ref. ^90^. The flexible amount of feeding guilds is inserted in each individual plot. Details on each ecosystem in Extended Data Table 4. Data after ref. ^90^. Source data

Source Data Extended Data Fig. 4 Generated by script Figure-Script-10.R.

**Extended Data Fig. 5:**
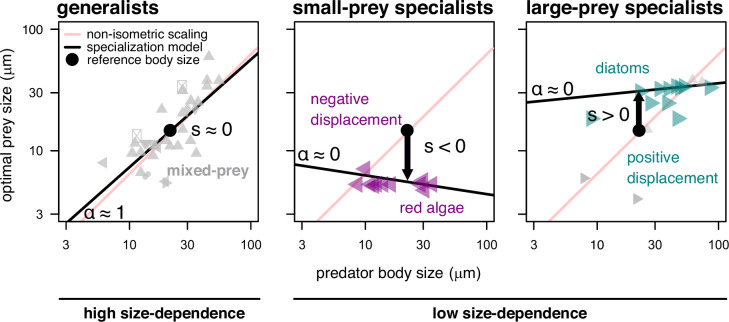
Effects of prey specialization on prey selection, exemplified for dinoflagellates. The specialization model describes the OPS as function of predator body size. Generalist predators have a neutral specialization (*s* = 0) and follow the allometric scaling (*α* ≈ 1). Small- and large-prey specialized predators instead have negative and positive specialization values, respectively, and nearly constant OPSs characterized by size independent prey selection (*α* ≈ 0). The specialization value is visualized as the displacement in the OPS for the reference body size. Data after ref. ^22^. Source data

Source Data Extended Data Fig. 5 Data from ref. ^22^ available at https://doi.org/10.1007/s00227-022-04102-2.

## Figures and Tables

**Extended Data Table 1:**
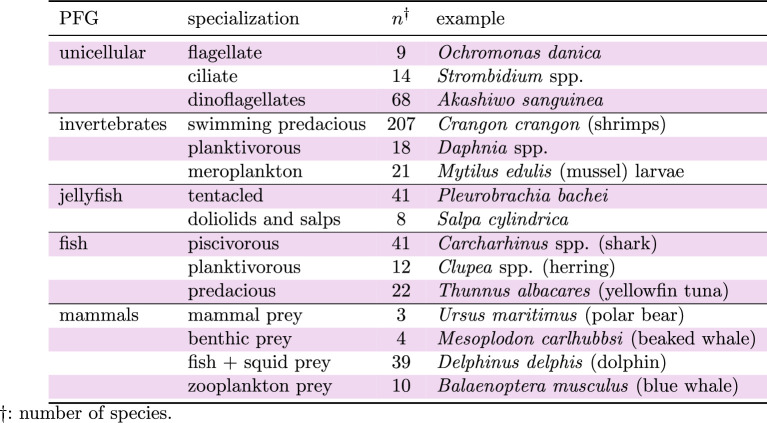
Predator Feeding Groups (PFGs), specialization guilds and number of observations (*n*)

**Extended Data Table 2:**
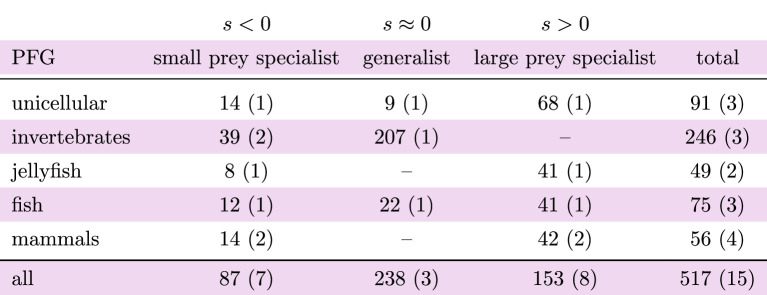
Distribution of species and guilds (in parenthesis) among prey selection strategies for all aquatic predators and each PFG

**Extended Data Table 3:**
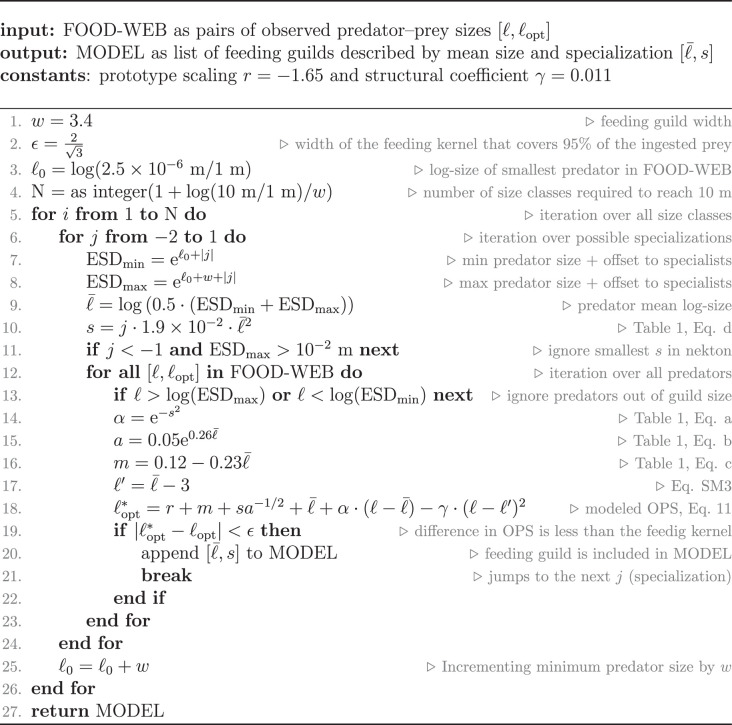
Food-webs assemblies using a mechanistic model

**Extended Data Table 4:**
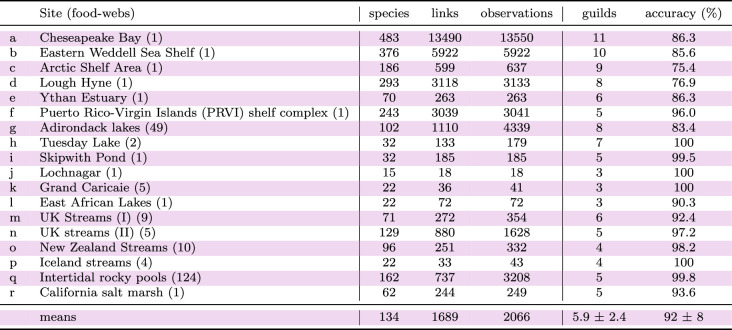
Model skill in reproducing real food-webs at 18 different sites

Species: number of identified trophic species, links: number of unique predator–prey pairs, observations: number of registered predator–prey interactions, guilds: number of feeding guilds utilized in our model, represented links: percentage of links correctly represented. See Fig. 5 for the modeling results, and Extended Data Fig. 3 for the site locations.

## Data Availability

Data required to reproduce analyses are available at https://github.com/ovgarol/aquatic-food-webs-Z and archived in Zenodo (ref. ^101^), which was compiled from several sources^17,22,25,27,87,88,90^. Taxonomy of included species are provided with this paper as a Supplementary Data file in CSV format. Source data are provided with this paper.

## Code availability

Custom scripts were used for simulation studies and data analyses. All code to generate figures is publicly available. Scripts to replicate analyses and figures are available at https://github.com/ovgarol/aquatic-food-webs-Z and archived in Zenodo (ref. ^101^).
