# Supplementary material for: The complex structure of aquatic food webs emerges from a few assembly rules
Source: Nat Ecol Evol. 2025 Feb 28;9(4):576–88. doi: 10.1038/s41559-025-02647-1 (PMC11976281; doi:10.1038/s41559-025-02647-1)
Supplement: Supplementary file 1 — Supplementary Methods 1–4 and Figs. 1–4. [file 41559_2025_2647_MOESM1_ESM.pdf]

nature ecology & evolution

Supplementary information https://doi.org/10.1038/s41559-025-02647-1

The complex structure of aquatic food webs emerges from a few assembly rules

In the format provided by the authors and unedited

Supplementary information: Complex structure of aquatic food-webs emerges from a few assembly rules

Ovidio García-Oliva[1,*] and Kai Wirtz[1,**]

[1]Helmholtz-Zentrum Hereon, Max-Planck-Straße 1, 21502 Geesthacht, Germany
[*]email: ovidio.garcia@hereon.de
[**]email: kai.wirtz@hereon.de

Supplementary Methods 1 Model parsimony

Optimal number of feeding guilds

We used a Gaussian mixture model to determine the underlying structure in the residuals r_i' of the observed OPS ($\ell_{\mathrm{opt},i}^{\mathrm{obs}}$) with the modeled using the allometric scaling adjusted for each PFG ($\ell_{\mathrm{opt},ki}^{\ddagger}$, Eq. 7):

$$r_i' = \ell_{\mathrm{opt},i}^{\mathrm{obs}} - \ell_{\mathrm{opt},ki}^{\ddagger}. \tag{S1}$$

We identified the optimal number of feeding guilds using the Bayesian Information Criterion (BIC) [1]. The distribution of residuals is better explained by a mixture model of three Gaussian distributions with equal variance ($\sigma = 0.66$, Fig. S1a) and means -1.85, 0.142, and 1.84. We pair these three distributions to the three components of the Z-pattern: one group of predators deviates negatively (small prey specialization), one positively (large prey specialization), and the last is centered in zero, thus following the allometric scaling (generalists) (Fig. S1b). Furthermore, combining these three groups defines the Z-pattern (Fig. S1c). The differences between the means (≈ 1.8) are greater than the feeding kernel that covers 99% of feeding interactions (three times the universal width of the feeding kernel $\frac{3}{\sqrt{3}} \approx 1.7$), thus likely depicting different prey selection patterns.

Relevance of trade-offs in food-web representation

To identify the most efficient food-web representation in terms of explanatory power and model uncertainty (complexity), we calculated the Akaike information criterion (AIC) for a series of models: (i) full-model, (ii) scaling of the Z-pattern is ignored (the stiffness is constant, and Eq. b in Table 1 was not used), (iii) rotation in the Z-pattern was ignored (the OPS scaling is either 0, -1, or 1, and Eq. a in Table 1 was not used), (iv) displacement in Z-pattern was ignored (the feeding mode is constant, and Eq. c in Table 1 was not used), (v) the specialization trait is ignored, but the PFGs are included (Eq. 7), and (vi) size only model (Eq. 6).

We used the formulation of AIC provided by [1]

$$\text{AIC} = n \cdot \log(\sigma^2) + \frac{2kn}{n-k-1}, \tag{S2}$$

where n is the number of observations, and k is the number of fitted parameters, and σ^2 is the reduced chi-squared statistic defined as

$$\sigma^2 = \frac{\sum_{i=1}^{n}\left[\ell_{\text{opt},i}^{\text{obs}} - \ell_{\text{opt},i}^{\text{mod}}\right]^2}{n-k-1}, \tag{S3}$$

where $\ell_{\text{opt},i}^{\text{mod}}$ is the OPS calculated by Eq. 11.

Our specialization model has the lowest AIC value (Fig. S2a), therefore, it is the most parsimonious. The inactivation of the trade-offs described in Table 1 does not improve the model parsimony (Fig. S2b-d). Despite its relative complexity, the specialization model outperforms simpler size-only models (Fig. S2e and f).

Supplementary Methods 2 Food-web topology and trophic level

The network topology, which denotes the arrangement of species (nodes) interconnected through predatory interactions (links), is a fundamental aspect of food-webs [2]. In empirical food-webs, this topology is summarized in the predation matrix A [3]. In this matrix, each row and column represents a species observed in the food-web, with each element a_{ji} equal to 1 if species j preys on species i, and 0 otherwise. However, comparing empirical networks directly with model reconstructions is impossible due to differences in their dimensions –i.e., the number of predator and prey species for observations, and the number of feeding guild in the model. To address this issue, we examine the relationship between body size and trophic level in both empirical and artificial food-webs to ensure that our model reproduces the trophic structure of the observations [4]. First, we define the predation matrix for artificially reconstructed food-webs a_{ji} using

$$a_{ji} = \begin{cases} 1 & \text{if } |\ell_{\text{opt}_j} - \bar{\ell}_i| < w \\ 0 & \text{otherwise,} \end{cases} \tag{S4}$$

where ℓ_{opt_j} represents the optimal prey log-size of predator guild j, $\bar{\ell}_i$ denotes the mean log-size of prey i, and w represents the feeding guild width.

Next, we establish the trophic levels of predator species in empirical and artificial food-webs. The trophic level T_j of species j is calculated as the average trophic level of its prey, plus 1, using

$$T_j = 1 + \frac{1}{K_j}\sum_i a_{ji}T_i, \tag{S5}$$

where $K_j = \sum_j a_{ji}$ is the number of prey of species j. Primary producers are considered basal species with $T_i = 1$. Here we use unweighted networks, where the strength

of predator–prey interactions is not considered [5]. We propose a more comprehensive representation of trophic levels using a randomized version of the predation matrix A', where $a'_{ji} = a_{ji} \cdot U(p)$. Here, $U(p)$ returns 1 if a randomly generated element from a uniform distribution in the interval $(0, 1)$ is greater than $p = 0.5$, allowing for the creation of more selective predators with higher p values.

Finally, we solve the trophic level (Eq. S5) using an ensemble of 30 randomly generated A' matrices for both empirical and artificial food-webs to compare their size-trophic level relationships. The trophic level of species j is represented by the ensemble mean $\bar{T}_j$. Both empirically and artificially assembled food-webs reproduce similar topologies, which follow similar trophic-level body size structure (Fig. S3).

Supplementary Methods 3 Additional model validation

The number of feeding guilds depends on the feeding guild width w as single fitting parameter of our model. The feeding width affects the accuracy and complexity of empirically assembled food-webs (Fig. S4). Generally, larger feeding guild width comes with lower number of required guilds but also lower accuracy of representing observed links, except for $3 < w < 4$, where accuracy is higher than the trend (Fig. S4a). We use a classical measure of food-web structure: the scaling behavior of connectance β (i.e., the number of realized trophic links divided by the number of potential links) as a function of the number of trophic species [6]. We conduct a sensitivity analysis by applying our model varying the feeding guild width w from 0.5 to 5 in 0.1 intervals. For each value of w, we calculated the accuracy and connectance, i.e., the number of realized links over the number of realized links [2] of reconstructed food-webs, as well as the scaling exponent of the connectance as a function of the number of feeding guilds using a log-log regression [7].

We use the exponent β to compare the complexity of reconstructed food-webs to the observations (Fig. S4b). The scaling exponent β of reconstructed food-webs is close to the one derived from observations around $w < 2$, and 3.4 (Fig. S4c). The best compromise of accuracy vs. complexity is at $w = 3.4$, which we use for the food-web reconstructions shown in Fig. 5.

The number of feeding guilds is related to the reconstructed food-web complexity, here expressed as the connectance [2] and its scaling exponent β [6]. We found that our reconstructions are more connected than the observed food-webs, as generally noted in other models [e.g. 8–10]. The higher connectance of our model reconstructions was due to a drastic reduction in the mean number of nodes from 134 species to 6 feeding guilds in observed and reconstructed food-webs, respectively. The modeled scaling exponent, however, is equal to the observed for the analyzed food-webs, suggesting that our approach preserves structural and topological properties of food-webs in general [e.g., stability 2, 6].

Supplementary Methods 4 Model parameter fit

Our model incorporates three groups of parameters, which are (1) general to all predators, (2) specific to a PFG, and (3) specific to a guild within a PFG. All sizes are represented as the natural logarithm of ESD expressed in meters, thus $\ell = \log(\mathrm{ESD}\ \mathrm{m}^{-1})$ and $\ell_{\mathrm{opt}} = \log(\mathrm{OPS}\ \mathrm{m}^{-1})$.

(1) As universal parameters, the prototype scaling r and the structural coefficient γ were estimated by a quadratic regression of Eq. S6 using the entire OPS data set.

$$\ell^{\dagger}_{\mathrm{opt},i} = r + \ell_i - \gamma \cdot \ell_i^2, \tag{S6}$$

where the subindex i represents individual species. Eq. S6 includes the effect of predator size and ignores the effects of PFG and specialization of the predator in the determination of the OPS. $\ell^{\dagger}_{\mathrm{opt},i}$ describes a size-only model that applies to any predator.

(2) For each predator group represented by the subindex k, we define the reference body size $\bar{\ell}_k$ equals the mean body log-size

$$\bar{\ell}_k = \frac{1}{n} \cdot \sum_{i\ \in\ \mathrm{group}\ k}^{n} \ell_i, \tag{S7}$$

where the summation is carried over all species belonging to the PFG k.

Two other group specific coefficients are defined as follows: the minimum body size ℓ'_k (reference for structural scaling) as 5% of the mean group size, which reads in log-scale

$$\ell'_k = \bar{\ell}_k - 3. \tag{S8}$$

and the group specific feeding mode as the mean of the log-PPSR ($= \ell_{\mathrm{opt}} - \ell$) corrected by the prototype and structural scaling:

$$m_k = r + \frac{1}{n} \cdot \sum_{i\ \in\ \mathrm{group}\ k}^{n} \ell_{\mathrm{opt},i} - \ell_i + \gamma \cdot (\ell_i - \ell'_k)^2. \tag{S9}$$

Combining Eqs. S9 and S6 we obtain a size scaling rule that accounts only effects of the PFG ($\ell^{\ddagger}_{\mathrm{opt},ki}$):

$$\ell^{\ddagger}_{\mathrm{opt},ki} = r + m_k + \ell_i - \gamma \cdot (\ell_i - \ell'_k)^2. \tag{S10}$$

This equation includes the effect of size and PFG of the predator and ignores the effect of the specialization in the determination of the OPS. Eq. S10 describes a OPS size model specific to each PFG.

(3) Alternatively to our approach we also tested a multiple regression model for OPS in each predator guild based on the theories proposed by [11, 12]. For each guild j within a predator group k, the OPS scaling exponent α_{kj} and intercept m'_{kj} result from log-log ordinary linear regression of reported body size and OPS,

$$\ell_{\mathrm{opt},kji} = \alpha_{kj} \cdot \ell_i + m'_{kj}. \tag{S11}$$

We also estimated the group specific stiffness a_k for the trade-off between size dependency and specialization displayed in Extended Data Fig. 1. For doing so, the guild specific specialization S_{kj} was calculated from the difference of the OPS expected by the linear regression (Eq. S11) and the OPS scaling specific to each PFG (Eq. S10). As both models have an explicit dependence in predator individual body size ℓ_i, we evaluated both equations in the reference body size, thus $\ell_i = \bar{\ell}_k$. This formalize our definition of specific specialization as

$$S_{kj} = \ell_{\mathrm{opt},kji} - \ell^{\ddagger}_{\mathrm{opt},ki} \quad \text{with } \ell_i = \bar{\ell}_k, \tag{S12}$$

thus

$$S_{kj} = m'_{kj} - r - m_k - (1 - \alpha_{kj}) \cdot \bar{\ell}_k \tag{S13}$$

The specific specialization, however, combines the effects of specialization and PFG. To disentangle these two components, we use a normalization constant, specific to each PFG, termed stiffness a_k. The stiffness a_k was evaluated by minimizing the deviation between the guild specific scaling exponent α_{kj} in Eq. S11 and the theoretical expectation Eq. 12

$$\min_{a>0} \left\{ \sum_{j \,\in\, \mathrm{group}\, k}^{n} \left[\mathrm{e}^{-a_k \cdot S_{jk}^2} - \alpha_{jk} \right]^2 \right\}. \tag{S14}$$

To compare the trade-off between guild specific size dependency (α_{kj}) and specialization (s_j) for different groups in Extended Data Fig. 1, the specific specialization S_{kj} was normalized using the stiffness of the respective group a_k

$$s_j = S_{kj} \cdot a_k^{1/2}. \tag{S15}$$

The uncertainties of the scaling exponents $\Delta\alpha_{kj}$ and specialization ΔS_{kj} were calculated from the standard errors of the ordinary linear regressions of OPS as function of predator body size of each guild (Eq. S11). $\Delta\alpha_{kj}$ was obtained directly from the regression, while the error of the specialization ΔS_{kj} was obtained from the first derivative of Eq. S13

$$\Delta S_{kj} = \Delta m'_{kj} + \Delta\alpha_{kj} \cdot \bar{\ell}_k, \tag{S16}$$

where $\Delta m'_{jk}$ is the standard error of the intercept of Eq. S11. In Eq. S16, the uncertainty of the structural coefficient was negligible ($\gamma = 0.011 \pm 0.001$), hence removed from the calculation. All calculations were performed with standard R.

References

[1] Richards, S. A. Testing ecological theory using the information-theoretic approach: Examples and cautionary results. *Ecology* **86**, 2805–2814 (2005). https://doi.org/10.1890/05-0074.

[2] Dunne, J. A., Williams, R. J. & Martinez, N. D. Food-web structure and network theory: The role of connectance and size. *Proc. Natl. Acad. Sci. USA* **99**, 12917–12922 (2002). https://doi.org/10.1073/pnas.192407699.

[3] Levine, S. Several measures of trophic structure applicable to complex food webs. *J. Theor. Biol.* **83**, 195–207 (1980). https://doi.org/10.1016/0022-5193(80)90288-X.

[4] Gauzens, B., Legendre, S., Lazzaro, X. & Lacroix, G. Food-web aggregation, methodological and functional issues. *Oikos* **122**, 1606–1615 (2013). https://doi.org/10.1111/j.1600-0706.2013.00266.x.

[5] Johnson, S., Domínguez-García, V., Donetti, L. & Muñoz, M. A. Trophic coherence determines food-web stability. *Proc. Natl. Acad. Sci. USA* **111**, 17923–17928 (2014). https://doi.org/10.1073/pnas.1409077111.

[6] MacDonald, A. A. M., Banville, F. & Poisot, T. Revisiting the links-species scaling relationship in food webs. *Patterns* **1**, 100079 (2020). https://doi.org/10.1016/j.patter.2020.100079.

[7] Murtaugh, P. A. & Derryberry, D. R. Models of Connectance in Food Webs. *Biometrics* **54**, 754–761 (1998). https://doi.org/10.2307/3109781.

[8] Li, J. *et al.* A size-constrained feeding-niche model distinguishes predation patterns between aquatic and terrestrial food webs. *Ecol. Lett.* **26**, 76–86 (2023). https://doi.org/10.1111/ele.14134.

[9] Gupta, A., Furrer, R. & Petchey, O. L. Simultaneously estimating food web connectance and structure with uncertainty. *Ecol. Evol.* **12**, e8643 (2022). https://doi.org/10.1002/ece3.8643.

[10] Albouy, C. *et al.* The marine fish food web is globally connected. *Nat. Ecol. Evol.* **3**, 1153–1161 (2019). https://doi.org/10.1038/s41559-019-0950-y.

[11] Warton, D. I., Wright, I. J., Falster, D. S. & Westoby, M. Bivariate line-fitting methods for allometry. *Biol. Rev. Camb. Philos. Soc.* **81**, 259–291 (2006). https://doi.org/10.1017/S1464793106007007.

[12] Costa, G. C. Predator size, prey size, and dietary niche breadth relationships in marine predators. *Ecology* **90**, 2014–2019 (2009). https://doi.org/10.1890/08-1150.1.

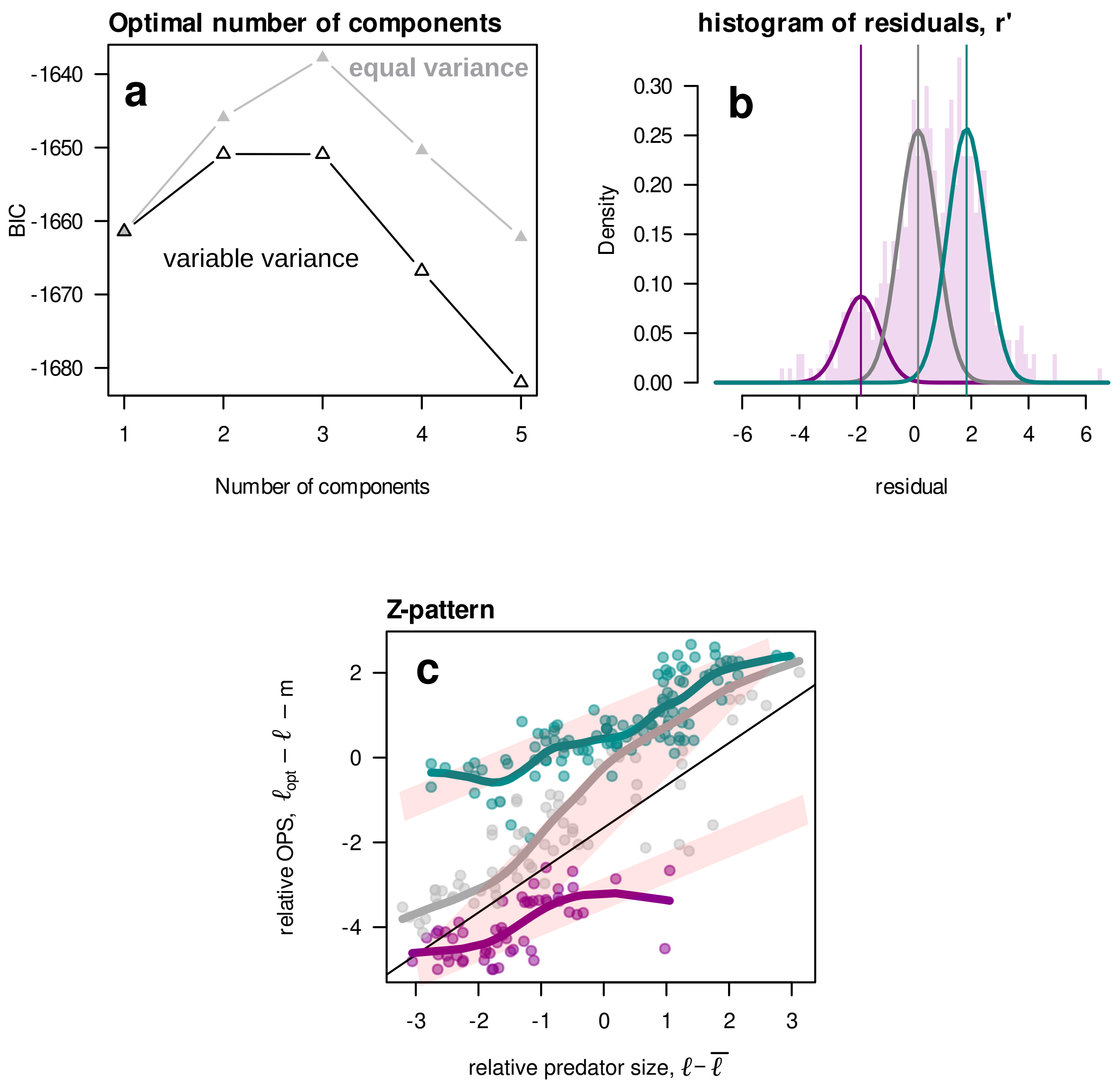

Supplement Figure S1: **Gaussian mixture model.** The optimal Gaussian mixture model (a) that explains the distribution of residuals (b) is the aggregation of three normal distributions with the same variance. This model roughly draws the Z-pattern (c). The colors of each observation were assigned by the Gaussian mixture model and the lines represent the cubic spline.

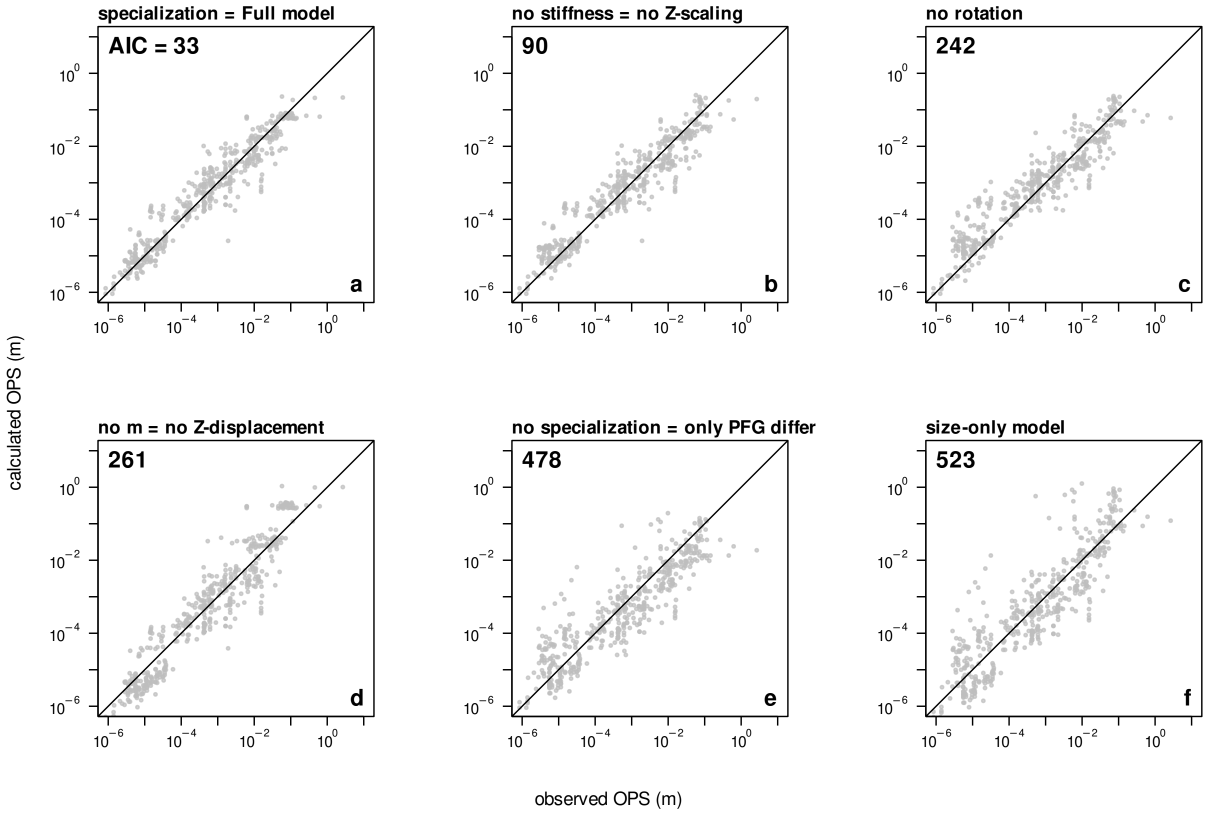

Supplement Figure S2: **Model parsimony.** The Akaike information criterion (AIC) for multiple model configurations.

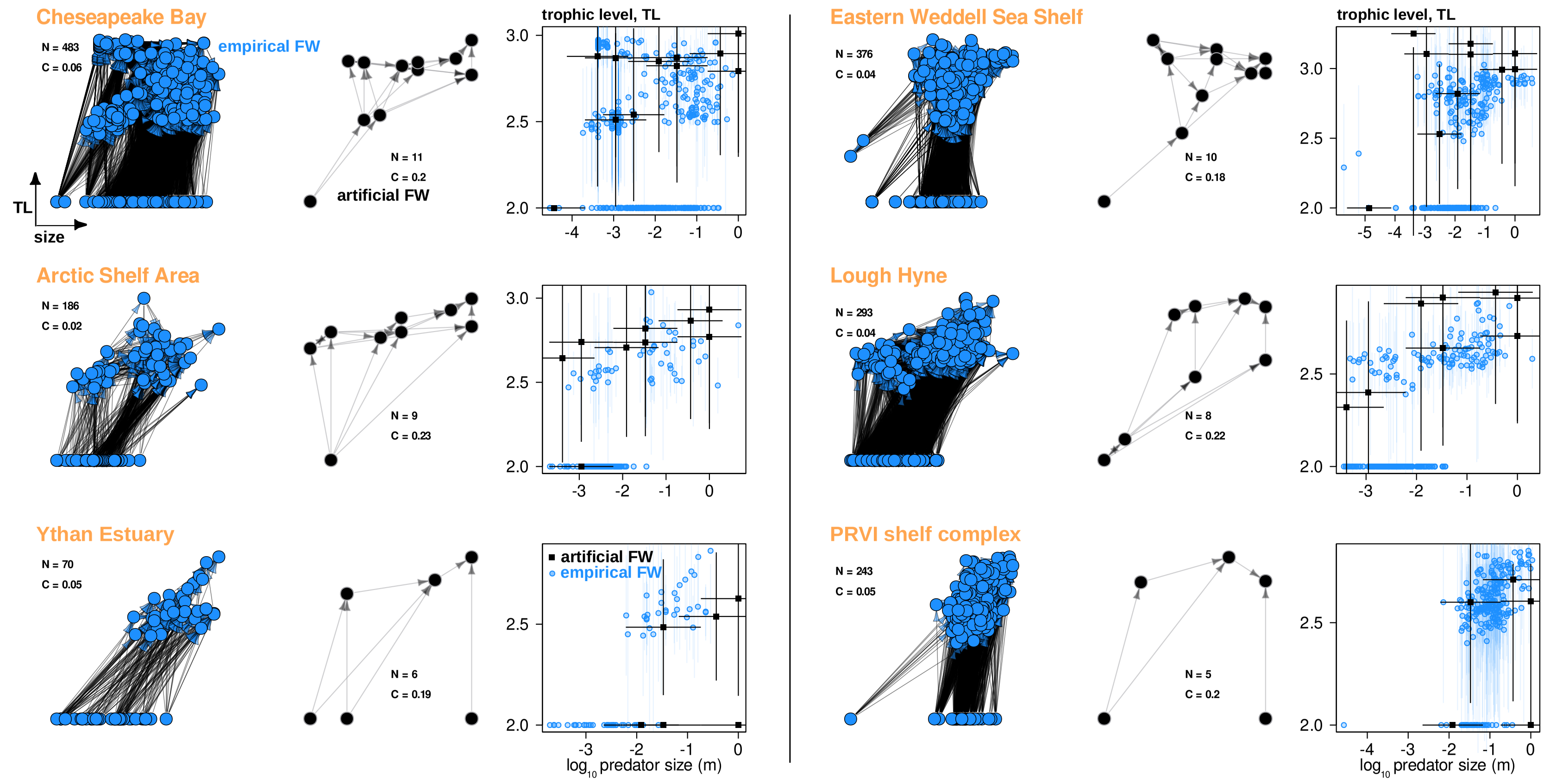

Supplement Figure S3: **food-web topology for six food-webs.** The topology and relationship of trophic level to body size are similar for empirically (blue) and artificially (black) assembled food-webs.

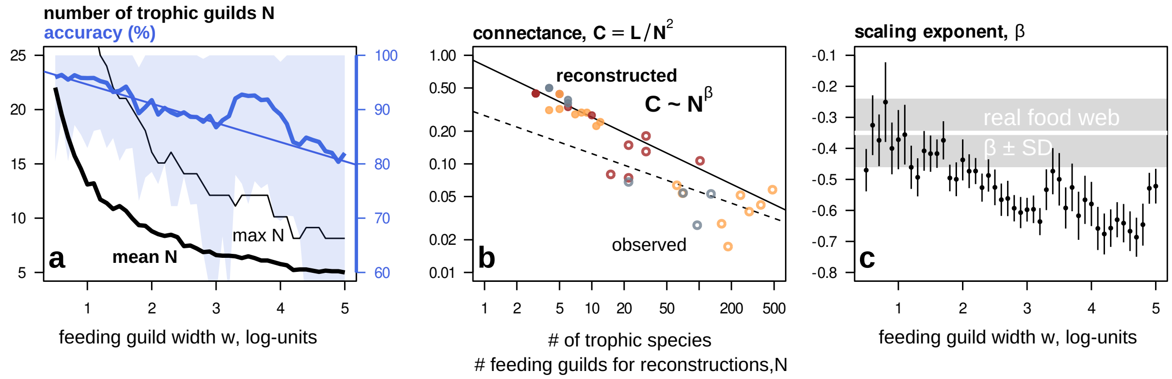

Supplement Figure S4: **Accuracy and complexity of artificial food-webs.** (a) Effect of feeding guild width w in the number of feeding guilds N (thin black line for maximum and thick black line for mean of the 18 ecosystems) and accuracy (solid blue line for the mean, light shade for the absolute range, and thin straight line for the trend excluding values in the range $3 < w < 4$). (b) Connectance C is a function of the number of trophic guilds N following a power-law for observed (void points, dotted line) and reconstructed food-webs when $w = 3.4$ (solid points, continuous line). The difference between observed and modeled β –the slope of the $\log N \sim \log C$ line– is not significant (log-log linear model with interaction, two-sided t-test $n = 18$, $t = -0.57$, $p > 0.1$), indicating no significant differences between observed and modeled β. (c) Complexity of reconstructed food-webs as described by the number of feeding guilds N and the scaling exponent of connectance β. The error bars are the standard errors of β obtained from log-log linear regressions of the 18 ecosystems. The observed β is given as white line, and the uncertainty –standard error of log-log regression of the observations dataset– as gray area ($n = 18$, $\beta = -0.35 \pm 0.11$).
